# Isolation and identification of *Candida* species in patients with orogastric cancer: susceptibility to antifungal drugs, attributes of virulence in vitro and immune response phenotype

**DOI:** 10.1186/s12879-016-1431-4

**Published:** 2016-02-23

**Authors:** Lourimar Viana Nascimento F. de Sousa, Vera Lúcia Santos, Andrea de Souza Monteiro, Marcus Vinicíus Dias-Souza, Sirlei Garcia Marques, Elaine Speziali de Faria, Elaine Alves de Oliveira Assunção, Simone Gonçalves dos Santos, Juan Moises Zonis, Daniel Gomes de Alvarenga, Rodrigo Assunção de Holanda, Jaqueline Gontijo de Sousa, Kênia Valéria dos Santos, Maria Aparecida de Resende Stoianoff

**Affiliations:** Laboratory of Mycology, Department of Microbiology, Institute of Biological Sciences, Federal University of Minas Gerais, Belo Horizonte, MG Brazil; Laboratory of Applied Microbiology, Department of Microbiology, Institute of Biological Sciences, Federal University of Minas Gerais, Belo Horizonte, MG Brazil; Laboratory of Microbiology, CEUMA University, São Luis, MA Brazil; Hospital of Federal University of Maranhão, São Luis, MA Brazil; Biomarkers Laboratory Diagnostics and Monitoring, Research Center René Rachou (CPqRR) - FIOCRUZ/MG, Belo Horizonte, MG Brazil; Laboratory of Microbiology, Vale do Rio Doce University, Governador Valadares, MG Brazil; Laboratory of oral and anaerobic microbiology, Department of Microbiology, Institute of Biological Sciences, Federal University of Minas Gerais, Belo Horizonte, MG Brazil; Oncology Core Experts, Governador Valadares, MG Brazil; Alvarenga Laboratory, Governador Valadares, MG Brazil; Laboratory of Basic and Applied Virology, Department of Microbiology, Institute of Biological Sciences, Federal University Minas Gerais, Belo Horizonte, MG Brazil; Laboratory of General Bacteriology and Clinical, Department of Pathology, Federal University of Espirito Santo, Health Science Center, Vitória, ES Brazil

**Keywords:** *Candida* spp, Orogastric cancer, Virulence, Antifungal, Immune phenotype

## Abstract

**Background:**

Because of the inherent immunosuppression of cancer patients opportunistic infections by *Candida* spp, occur frequently. This study aimed to identify *Candida* species in the oral mucosa of 59 patients with orogastric cancer (OGC) and to analyze the immunological phenotype of these patients.

**Methods:**

The yeasts were identified by MALDI-TOF mass spectrometry (MS). For all isolates, we performed phospholipases and proteinases assays, in vitro adherence to buccal epithelial cells (BEC), minimum inhibitory concentration of antifungal drugs and determined the cytokine profile by Cytometric Bead Array flow citometry assay.

**Results:**

*C. albicans* was the most prevalent species in OGC patients (51.6 %) and control group (66.7 %). *Candida* spp. strains isolated from OGC patients exhibited better adherence to BEC (*p* = 0.05) than did the control group. Phospholipases production by *Candida* strains from OGC patients was lower (51.6 %) than in the control group (61.9 %). Proteinases were detected in 41.9 % and 4.8 % of the yeasts from OGC patients and control group, respectively. Significant differences were found in the serum of OGC patients compared to the control group for IL-2, IL-10, TNF-α, IFN-γ and IL-17.

**Conclusions:**

The results of this work suggest increased virulence of yeasts isolated from OGC patients and, that this may interfere with the immune phenotype*.*

## Background

Malignant neoplasms or cancer is defined as a chronic degenerative disease of rapid and abnormal growth of defective cells, which may spread through the body (metastasis) and increase the rate of mortality [1]. The process of carcinogenesis and oncogenesis comprises early changes in DNA to tumor formation, which may cause slow and progressive destruction of the host organism [2].

Cancer patients with metastasis have a high probability of mucocutaneous and bloodstream infections by fungi, including *Candida* species. Candidiasis is a fungal infection of extensive spectrum, which affects mainly immunocompromised patients. *Candida* species of medical interest, such as *Candida albicans* to non-*albicans, C. glabrata, C. tropicalis, C. krusei* and *C. parapsilosis*, can be found commensally in the microbiota of human cavities (such as rectal, oral, vaginal, urethral, nasal, and aural) and on skin, although their presence in the microbiota of healthy people remains unexplained. However, nutritional factors, interactions with the microbiota, and the presence of salivary antibodies have been suggested as possible influencing factors in the incidence of these yeasts, which are usually controlled by local factors, such as competition with bacterial cells of the resident microbiota [3–6].

*Candida albicans* is a dimorphic fungus that is part of the commensal microbiota of the oral cavity. When the host immune defenses are impaired or when the normal microbiota is disturbed, *C. albicans* triggers recurrent infections of the oral mucosa and tongue [3]. The yeast’s colonization and its ability to be an opportunistic pathogen make candidiasis common worldwide [3]. For cancer patients, in addition to the intrinsic factors related to treatment such as placement of central venous catheters and receipt of parenteral nutrition, there are additional risk factors for candidemia. In these individuals, there may exist chemotherapy-induced neutropenia and/or mucositis and radiation-induced tissue injury, which facilitate fungemia by *Candida* spp. [7, 8].

The pathogenicity of *Candida* spp. is related to a combination of factors contributing to their virulence, such as their ability to grow at 37 °C, their phenotypic variability (switching), their transition from budding-to-hyphae or pseudo-hyphae and biofilm formation. This pathogenicity is also related to the presence of homologous molecules, such as the human integrin CR3 receptor, which facilitates adherence to epithelial cells, and the ability to produce hydrolytic enzymes, such as phospholipases, aspartyl proteinases (Sap), and lipases [9, 10]. The adherence to the mucosal surface of the host probably occurs by the interaction between the microorganisms’adhesins and receptors on epithelial cells [11]. Secretion of phospholipases is considered a key attribute for the invasion of host mucosal epithelium, by disrupting the phospholipid bilayer, which is amajor component of all cell membranes [9].

Oropharyngeal candidiasis is the most common fungal infection that affects cancer patients. Particularly, patients in radiotherapy for head and neck cancer are more susceptible to colonization by *Candida.* It was described in murine models that mucocutaneous infections are usually associated with deficient cellular immune responses, and systemic infections are more related to innate immunity, characterized by impairments of neutrophil recruitment and activity [12, 13].

The guidelines of the American Society of Infectious Diseases recommends treatment with caspofungin and fluconazole for patients who had not been previously exposed to azoles or amphotericin B. The combined therapy of amphotericin B with fluconazole is recommended for the initial treatment of chronic candidemia, and 5-fluorocytosine combined with either of these drugs might be recommended [14]. The most relevant antifungal commercially available has similar pharmacodynamics, the fungal membrane sterols being the main targets [15].

There are different inflammatory responses in the tumor microenvironment, this contains the innate immune response cells, also found cells of the adaptive immune response, and cancer cells surrounding by stroma [16]. In this microenvironment, there are different interactions through cytokines, chemokines, and even interactions through direct contact, by interaction of cell receptors to activate or induce autocrine and paracrine pathways, in order to control the shape and tumor expansion. However, the expression of several mediators and modulation of immune response as well as the amount and state of activation of different types of cells in the tumor microenvironment that dictate whether the local inflammation will promote tumor growth or whether an immune response suppression cancer cells will follow [17, 18]. Patients with cancer present clinical cytokine patterns that suggest that simultaneous immunostimulation and immunosuppression occur in patients with cancer, with increased concentrations of the cytokines like TNFα, interleukin 6, interleukin 8, interleukin 10, and interleukin 18 [19]. Nevertheless, it was suggested that one specific cytokine pattern may have a prognostic effect, since high interleukin 6 or interleukin 10 serum concentrations are associated with negative prognoses in independent types of cancer [16].

The immune mechanisms of defense against fungal infections are numerous, and range from protective mechanisms that were present early in evolution (innate immunity) to sophisticated adaptive mechanisms that are induced specifically during infection and disease (adaptive immunity). The first-line innate mechanism is the presence of physical barriers in the form of skin and mucous membranes, which is complemented by cell membranes, cellular receptors and humoral factors. There has been a debate about the relative contribution of humoral and cellular immunity to host defense against fungal infections. *Candida albicans*, part of the normal microbial flora associated with mucous surfaces, can be present as congenital candidiasis or as acquired defects of cell-mediated immunity. Resistance to this yeast is associated with Th1, whereas Th2 immunity is associated with susceptibility to systemic infection [20].

Therefore, this study aimed to identify *Candida* species in the oral mucosa of patients with orogastric cancer and to analyze the immunological phenotype of these patients. Yeast strains were identified by standard taxonomic keys and Matrix Assisted Laser Desorption Ionization – Time Of Flight – Mass Spectrometry (MALDI-TOF). Phenotypic methods were employed for identifying virulence factors, and antifungal susceptibility testing was performed through broth microdilution. Furthermore, Cytometric Bead Array (CBA) flow citometry was used for the simultaneous detection and quantification of IL-2, IL-4, IL-6, IL-10, IL17, TNF-α and IFN-γ.

## Methods

### Patient selection and biological samples

The consent of the patients and the control group was made in writing, through the TIC (Term of informed consent) after this study is approved by the Research Ethics Committee of the University of Vale do Rio Doce (COEP PQ25 / 10-10). Fifty nine patients with orogastric cancer were selected. Cancer diagnosis was confirmed by histopathology assays, and thirty-four healthy individuals were selected for the control group. The control group comprised health professionals, dentists, and teachers from Univale University. These individuals were invited because this group should be made of people who did not show any alterations in the oral cavity, especially regarding the presence of carcinomas. These selected patients were free of any type of disease. The group of individuals with cancer had not started chemotherapy by the time of the commencement of the study. However, these individuals had positive diagnosis for cancer, confirmed by histopathological examination. Both groups were evaluated for the presence of *Candida spp*. The collection of the samples was performed by specialist physicians of Governador Valadares city (MG). Inclusion criteria for patients comprised a previous diagnosis of advanced cancer and not being subjected to chemotherapy or radiotherapy. Clinical specimens for microbiological analysis were collected using sterile swabs and placed in tubes containing Sabouraud dextrose broth (Difco®) with chloramphenicol (0.1 mg/mL), and these specimens were sent to the laboratory and incubated at 28 °C for 72 h for subsequent microbiological testing. Direct examination was not performed for the presence of pseudohyphae or fungal cells.

### Isolation and identification of strains

The tubes containing yeasts were incubated without shaking in an incubator at 37 °C, disposed in galleries. Yeast strains were isolated after inoculation of positive samples in Sabouraud dextrose agar (SDA, Difco) *Candida*. The yeast isolates were inoculated in Chromagar*Candida* (Difco Laboratories, Detroit, MI) to obtain pure cultures and for morphological identification according to standard taxonomic keys [21, 22]. The tests used in the differentiation of *Candida* species were: (1) presence of growth at 42 and 45 °C [23], characteristic observed only for the *C. albicans*, (2) presence of growth on the hypertonic Sabouraud broth (NaCl 6.5 %) [24], characteristic observed only for the *C. albicans*; (3) Also, slide cultures were prepared to visualize the difference in the production of chlamydospores.

### Slide culture and microscopy

A fragment of cornmeal agar prepared with Tween 80 was placed on a sterile glass slide. The yeast cells were inoculated by making the three central grooves in the agar fragment. The inoculum was covered with a sterile cover slip and incubated in sterile petri dish with a piece of cotton soaked in sterile water at 28 °C for approximately seven days. Yeast growth was analyzed after five days of incubation to study the formation of pseudohyphae, hyphae and chlamydospores to make the morphological identification.

### Identification of yeasts by MALDI-TOF MS

The confirmation of *Candida* species by Matrix-Assisted Laser Desorption Ionization – Time-of-Flight Mass Spectrometry (MALDI-TOF MS) was performed using the VITEK–MS system (bioMérieux, Marcy-l’Etoile, France). Yeast cells were grown on SB agar medium plates for 24 h, at 37 °C. A loopful of yeast cells was directly transferred from the culture medium onto each position of the disposable target slides (bioMérieux, France), which were inoculated with a small amount of a single yeast colony to provide a thin layer of organism using a disposable plastic loop. Afterwards, 1 μL of 70 % formic acid was added to each sample on the target slide and allowed to air dry it before adding 1 μL of α-cyano-4-hydroxycinnamic acid matrix solution. Finally, the mass spectra acquired for each isolated yeast were compared to the known mass spectra contained in the database and given a confidence score according to how closely the acquired spectra matched those contained in the database. The resulting slides were then analyzed in the VITEK® MS instrument, using the automatic database analysis of the obtained mass spectra within MYLA® software (bioMérieux, France) to provide isolate identification.

### Virulence factors

#### Hydrolytic enzymes

Sixty-two isolates of *Candida* spp. isolated from the oral cavity of patients with orogastric cancer and 21 isolates from the oral mucosa of individuals in the control group were selected for this assay. The methods we used to determine proteinases activity were recommended by Cassone et al. [25] and De Bernardis et al. [26]. Proteinases secretion was evaluated on solid medium containing 11.7 g of Yeast Carbon Base (Difco Laboratories,Detroit, MI, USA), 1.0 g of yeast extract, and 2.0 g of bovine serum albumin (BSA, Merck Sharp & Dohme, Kenilworth, NJ, USA)/100 mL of water. The pH was adjusted to 5.0, medium was sterilized by filtration and added to an agar solution sterilized by autoclaving (agar 18 g in 900 ml of distilled water). The inoculum consisted of a suspension of 10^6^ cells/mL deposited on the agar. The plates were incubated for seven days at 28 °C and proteolysis of BSA was visualized as a clear halo after staining with amido black staining (1 g black starch, 199 mL glacial acetic acid, 100 mL distilled water). Proteinases activity was classified as negative if there were no visible clarification, 1+ when proteolysis surrounding the colony ranged from 1 to 2 mm, and 2+ when discoloration of agar exceeded 3-5 mm around the colony. The phospholipases production was verified using the egg yolk agar plate method according [27]. The Petri dishes were incubated at 37 °C, and the diameters of the colonies with the zones of precipitation were measured after 7 days of incubation. The zone of phospholipases (PZ) was calculated as the colony diameter divided by the diameter of the colony plus the precipitation zone. The production of protease was demonstrated by clear halos around the colonies on agar plates containing BSA. The PZ index was determined in the same way as for phospholipases. Each isolate was tested in duplicate.

### Adhesion of Candida strains to BEC

For adhesion studies of yeasts in buccal epithelial cells, we used 26 isolates (*C. albicans, C. tropicalis, C. glabrata* and *C. krusei*) from cancer patients and 10 yeasts from patients in the control group (*C. albicans, C. parapsilosis* and *C. glabrata*). The yeast cells were pre-incubated in SDA plates and incubated at 37 °C for 24 h before the test to obtain the inoculum. The assay was performed as described by Machado et al [28]. Next, 3 colonies were transferred to 40 mL of Sabouraud broth (Difco). After incubation at 37 °C for additional 24 h, the yeasts were Gram stained in order to verify the purity of the suspension. Next, the cells were centrifuged (3000 g; 15’) and washed 3 times in 15 mL of saline phosphate buffer (PBS; pH 7.4). A suspension containing 10^7^ cells/mL was obtained in a Neubauer chamber (Laboroptik, Friedrichsdorf, Hesse, Germany) using the Trypan blue exclusion method.

The assay was performed as described by Kimura and Pearsall [29] Human buccal cells (HBECs) were harvested from a healthy young adult non-carrier of *Candida* yeasts in the oral cavity with two sterile swabs and added to 10 mL PBS. The BEC suspension was washed four times and centrifuged to remove adherent microorganisms (3000 *g,* 10 min). The suspension of the yeasts was then diluted to a concentration of 10^5^ cells/mL, counted with a Neubauer chamber and used immediately. For the test, 0.5 ml of the BEC suspension (1 x 10^5^ cell/ml) and 0.5 ml of the yeast suspensions (1 x 10^7^ cell/ml) were mixed gently and incubated at 37 °C for 1 hourunder shaking (40 rpm).

The cells were then harvested on polycarbonate membranes with an 8-μm pore diameter (*Sartorius*-Stedim, Biotech) and washed with PBS to remove non-adhering yeast. The filter was removed with the aid of forceps and pressed against a glass slide. After 10 s, the filter was gently removed, leaving the BEC adhered to the glass slide. The preparation was dried, fixed by heat and stained with crystal violet. The number of yeasts per BEC 50 was quantified by optical microscopy. Folded or overlapping cells were excluded. After counting, the mean and standard deviation of each strain weretaken.

### Antifungal susceptibility testing

Susceptibility to fluconazole, izoconazole, voriconazole, ketoconazole and amphotericin B were performed according to the M27-A3 document of the CLSI [30]. Fluconazole (Pfizer, São Paulo, Brazil), isoconazole, voriconazole, ketoconazole (Jansen Pharmaceutica, Beerse, Belgium) and amphotericin B (Sigma, St Louis, Millstone, USA) were obtained as reagent grade powders. Stock solutions were prepared in dimethylsulfoxide (amphotericin B) or water (fluconazole, isoconazole, voriconazole and ketoconazole). Serial two-fold dilutions were prepared, and dilutions were made in RPMI 1640 medium (Sigma) buffered to pH 7 with 0.165 M morpholinopropanesulfonicacid (Sigma). Aliquots (100 μL) of each agent at a two-fold final concentration were dispensed into the wells of plastic microdilution trays. The trays containing fluconazole, isoconazole, voriconazole and ketoconazole were sealed and frozen at -70 °C until used. Amphotericin B was prepared immediately before use.

The inoculum suspension was adjustedwith a *spectrophotometer* (660 nm) to a final density of 1.5 ± 1.0 x10^3^ cells/ml. Final concentrations of the antifungal agents ranged between 0.03 and 64 μg/ml. The trays were incubated at 35 °C and the MIC (Minimum Inhibitory Concentration) endpoints were read after 48 h of incubation. Drug-free and yeast control wells were included.

Following incubation, the MICs of fluconazole, isoconazole, voriconazole and ketoconazole were read as the lowest concentration at which a prominent decrease (approximately 80 %) in turbidity was observed compared to the drug-free control. The MICs of amphotericin B were read as inhibition of 100 % of the growth. The interpretative criteria for the susceptibility to fluconazole, isoconazole, voriconazole and ketoconazole were used as described elsewhere [15, 30]. No interpretative criteria were defined for amphotericin B; however, for comparison, isolates inhibited by concentrations <1.0 μg/ml were considered susceptible.

### CBA analysis of cytokines

The determination of cytokines in the serum from 33 cancer patients (randomly selected) and 31 from control individuals was determined using the human Th1/Th2 cytokine cytometric bead array (CBA) kit (BD PharMingen, San Diego, CA). Cytokines IL-2, IL-4, IL-6, IL-10, IL-17, TNF-α and IFN-γ were detected simultaneously by adding 50 μL of each sample mixed with 50 μL of mixed capture beads and 50 μL of the human Th1/Th2 PE detection reagent consisting of PE-conjugated anti-human cytokines. The samples were incubated at room temperature for 3 h in darkness. After incubation with the PE detection reagent, samples were washed once and resuspended in 300 μL of wash buffer before acquisition on the FACSCalibur (BD Biosciences, Sunnyvale, CA). Data were analyzed using CBA software (BD PharMingen). Standard curves were generated for each cytokine using the mixed cytokine standard provided by the kit. The concentration for each cytokine in cell supernatants was determined by interpolation from the corresponding standard curve. The range of detection was 20–5000 pg/mL for each cytokine measured by CBA.

### Statistical analysis

Analysis of variance (ANOVA) was performed using the process of Minimum Significant Difference (MSD) for comparison of means. Student t testwas used to compare means, where *p*values ≤ 0.05 were considered statistically significant. Calculations were performed using EPISTAT (TL Gustafson, Round Rock, USA) software.

## Results

### Study group

Approximately 50 (85 %) cancer patients were positive for culture of *Candida* species in the oral mucosa. In the control group, 20 patients showed strains of *Candida* in theoral cavity (59 %). Of the 50 individuals in the study group (orogastric cancer), there were21 (42 %) with oral cancers, 14 (28 %) with esophageal cancer, 2 (4 %) with neck cancer, 7 (14 %) with stomach cancer, and 6 (12 %) with laryngeal cancer.

A total of 83 *Candida* isolates representing 6 different species were isolated from patients with orogastric cancer and individuals without cancer (control group). All patients in this study (control and test groups) had no clinical signs and no symptoms of candidiasis. However, they were colonized by yeasts of the gender *Candida*.

All isolates were identified by MALDI-TOF MS. In the group of cancer patients, 32 (51.6 %) isolates were identified as *Candida albicans*, 4 (6.5 %) as *C. tropicalis*, 9 (14.5 %) as *C. glabrata*, 4 (6.5 %) as *C. krusei*, 8 (12.9 %) as *C. parapsilosis* and 5 (8 %) as *C. lusitaniae*. The control group included 14 (66.7 %) isolates identified as *C. albicans*, 5 (23.8 %) as C. *parapsilosis*, 1 (4.8 %) as *C. krusei* and 1 (4.8 %) as *C. glabrata*. Some patients have more than one species of *Candida* in the oral cavity, while not presenting clinical manifestation of candidiasis. Patients with more than one yeast are YCa 36, 37 e 38 – C. *albicans, C. parapsilosis* and *C. glabrata* and YCa 48, 49 and 50 – *C. albicans, C. parapsilosis* and *C. lusitaniae*. YCa 22 and 23 – *C. albicans* and *C. krusei*, YCa 40 and 41 – *C. glabrata* and *C. lusitaniae*, YCa 42 and 43 – *C. glabrata* and *C. lusitaniae*, YCa 44 and 45 – *C. parapsilosis* and *C. albicans*, Yca 46 and 47 – *C. albicans* and *C. glabrata*, YCa 54 and 55 – *C. albicans* and *C. parapsilosis*, YCa 56 and 57 – *C. lusitaniae* and *C. glabrata*, YCa 58 and 59 – *C. albicans* and *C. lusitaniae*.

### Adhesion, phospholipases and proteinases

The *Candida* strains isolated from the oral cavity of subjects with cancer were more adherent than those of the oral cavity of people without alteration of the mucosa (*p* ≤ 0.05). The group with cancer had the double of the number of adhered yeast cell compared to the control group. The median number of yeast to 430 CEB was 2 in yeast strains from patients with cancer. In *Candida* strains isolated from individuals with normal oral mucosa (control group), this value was 1. Minimum values were zero for both groups and the maximum values were 21 and 18 for the cancer and control groups, respectively. The statistical analysis for nonparametric data showed differences between the two groups (*p* ≤ 0.05).

The PZ value was determined as the ratio of the diameter of the colony to the total diameter of the colony plus the precipitation zone, and it was scored and categorized as follows: PZ value = 1 (negative); PZ value = 0.75-0.9 (low producers); PZ value = 0.51-0.74 (moderate producers); and PZ value = 0.35-0.5 (high producers) [31]. For phospholipases production, 54.2 % of yeast strains produced the enzyme on solid medium. In addition, 32.5 % of the yeast strains produced proteinase. Only 13.3 % of the isolates were positive for the production of phospholipases and proteinases (Table 1).

**Table 1:** Proteinases and phospholipases activity of *Candida* spp. isolates from patients with orogastric cancer and healthy individuals (control group)

Isolates		Phospholipase	Proteinase
YCa1	*C. albicans*	N	N
YCa2	*C. parapsilosis*	N	N
YCa3	*C. parapsilosis*	HP	N
YCa4	*C. glabrata*	HP	HP
YCa5	*C. albicans*	N	N
YCa6	*C. albicans*	HP	N
YCa7	*C. albicans*	HP	N
YCa8	*C. albicans*	HP	N
YCa9	*C. albicans*	HP	HP
YCa10	*C. albicans*	N	N
YCa11	*C. albicans*	N	N
YCa12	*C. albicans*	N	N
YCa13	*C. albicans*	HP	N
YCa14	*C. albicans*	HP	PO
YCa15	*C. tropicalis*	HP	HP
YCa16	*C. albicans*	N	HP
YCa17	*C. glabrata*	HP	HP
YCa18	*C.albicans*	PO	N
YCa19	*C. glabrata*	N	P
YCa20	*C. albicans*	HP	N
YCa21	*C. glabrata*	N	PO
YCa22	*C. albicans*	N	N
YCa23	*C. krusei*	HP	HP
YCa24	*C. albicans*	N	N
YCa25	*C. tropicalis*	HP	PO
YCa26	*C. krusei*	N	N
YCa27	*C. glabrata*	N	HP
YCa28	*C. parapsilosis*	HP	HP
YCa29	*C. tropicalis*	N	N
YCa30	*C. albicans*	HP	N
YCa31	*C. albicans*	HP	HP
YCa32	*C. parapsilosis*	HP	HP
YCa33	*C. albicans*	HP	N
YCa34	*C. albicans*	HP	N
YCa35	*C. tropicalis*	N	N
YCa36	*C. albicans*	HP	N
YCa37	*C. parapsilosis*	N	N
YCa38	*C. glabrata*	N	HP
YCa39	*C. krusei*	N	HP
YCa40	*C. glabrata*	N	HP
YCa41	*C. lusitaniae*	N	HP
YCa42	*C. albicans*	HP	N
YCa43	*C. lusitaniae*	N	PO
YCa44	*C. albicans*	HP	N
YCa45	*C. parapsilosis*	N	HP
YCa46	*C. albicans*	HP	N
YCa47	*C. glabrata*	N	HP
YCa48	*C. albicans*	HP	N
YCa49	*C. parapsilosis*	N	HP
YCa50	*C. lusitaniae*	N	N
YCa51	*C. krusei*	N	HP
YCa52	*C. albicans*	HP	N
YCa53	*C. albicans*	HP	N
YCa54	*C. albicans*	HP	N
YCa55	*C. parapsilosis*	HP	HP
YCa56	*C. lusitaneae*	N	HP
YCa57	*C. glabrata*	N	HP
YCa58	*C. albicans*	N	N
YCa59	*C. lusitaniae*	N	N
YCa60	*C. albicans*	HP	N
YCa61	*C. albicans*	HP	N
YCa62	*C. albicans*	HP	N
YCo2	*C. albicans*	HP	N
YCo3	*C. parapsilosis*	N	N
YCo4	*C. parapsilosis*	N	N
YCo5	*C. albicans*	HP	N
YCo7	*C. albicans*	HP	N
YCo11	*C. albicans*	HP	N
YCo13	*C. albicans*	N	N
YCo16	*C. glabrata*	N	N
YCo17	*C. parapsilosis*	N	N
YCo9	*C. albicans*	HP	N
YCo23	*C. para*	N	N
YCo26	*C. albicans*	HP	N
YCo28	*C. albicans*	HP	N
YCo33	*C. para*	N	N
YCo35	*C. albicans*	HP	N
YCo36	*C. albicans*	HP	N
YCo37	*C. albicans*	HP	N
YCo39	*C. albicans*	HP	HP
YCo42a	*C. albicans*	HP	N
YCo42b	*C. krusei*	N	N
YCo43	*C. albicans*	HP	N

*YCa* yeasts from patientes with cancer, *YCo* yeasts from control group, *HP* high positive, *PO* Positive

### Susceptibility assays

A total of 83 isolates of *Candida* spp. were analyzed for their susceptibility to ketoconazole (KET), fluconazole (FLU), isoconazole (ISO), amphotericin B (AmpB), and voriconazole (VOR). The results of these assays are presented in Table 2. MIC values were determined after 24 h of incubation, as most yeast had a strong increase in the MIC from 24 to 48 h, with a high growth, called trailing. Interpretive criteria for susceptibility to fluconazole and isoconazole were adopted as published by Rex et al. [32], and Fothergill et al. [33]. We considered strains susceptible when MIC was < 8.0 μg/mL for these drugs. For voriconazole, interpretive criteria for susceptibility of the CLSI (2008) were adopted. We considered yeast strains susceptible when the MIC was < 1.0 μg/mL. Interpretation criteria for amphotericin B were determined following the standards adopted by the CLSI [19]: yeast strains were considered susceptible when the MIC was < 1.0 μg/mL. The results are presented in Table 2.

**Table 2:** Susceptibility profile of *Candida* spp*.* isolates from patients with orogastric cancer and healthy individuals (control group)

Antifungal drugs	Species	Cancer orogastric patients				Control group			
		MIC_90_ 24 h	Range MIC 24 h	MIC _90_ 48 h	Range MIC 48	MIC_90_ 24 h	Range MIC 24 h	MIC _90_ 48 h	Range MIC 48
		Concentration (μg/mL)				Concentration (μg/mL)			
	*C. albicans*	32^d^	0.125–32	64^d^	1.0 – 64	32^a^	0,25–32	64^d^	0.5–64
FLU	*C. glabrata*	8.0^d^	0.5– 32	32^d^	8.0 – 64	0.25^a^	0.25	2.0^a^	2.0
	*C. parapsilosis*	8.0^a^	0.5–16	16^d^	8.0–64	1.0^a^	0.5–4.0	8.0^a^	0.5–32
	*C .krusei*	4.0^a^	0.5–32	0.5^a^	0.25–64	0.25^a^	0.25	0.25^a^	0.25
	*C. tropicalis*	64^d^	1.0–64	64^d^	32–64	-	-	-	-
	*C. lusitaniea*	8.0^a^	4.0–8.0	64^d^	64	-	-	-	-
	*C. albicans*	8.0^a^	0.0625–32	16^d^	0.25–64	4.0^a^	0.125 –32	32^d^	0.25–64
	*C. glabrata*	0,5^a^	0.25–4	4.0^a^	1.0– 32	0	0	2.0^a^	2.0
ISO	*C. parapsilosis*	1.0^a^	0.0625–1.0	2.0^a^	0.5–8	2.0^a^	0.5–2.0	.0^a^	1.0–32
	*C. krusei*	0	0–32	4.0^a^	0–32	0	0	0	0
	*C. tropicalis*	4.0^a^	0.125–16	8.0^a^	2.0–32	-	-	-	-
	*C. lusitaniea*	0	0–2.0	8.0^a^	1.0–16	-	-	-	-
	*C. albicans*	64^d^	0.0625–64	64^d^	0.0625–64	2.0^d^	0.125–2.0	64^d^	0.25–64
	*C. glabrata*	2.0^d^	0.25–2.0	4.0^d^	4.0 – 64	0	0	0.25^a^	0.25
VOR	*C. parapsilosis*	2.0^d^	0.25–2	4.0^d^	1.0–64	0.125^a^	0–0.5	2.0^d^	0.25–64
	*C. krusei*	0	0–8.0	2.0^d^	0–16	0	0	0	0
	*C. tropicalis*	32^d^	0.5–64	64^d^	32–64	-	-	-	-
	*C. lusitaniea*	1.0^a^	0–16	16^d^	1.0–64	-	-	-	-
	*C. albicans*	0.5^a^	0.125–32	0.5^a^	0.125–32	0.5^a^	0.25–0.5	0.5^a^	0.25–0,5
	*C. glabrata*	0.5^a^	0.25– 0.5	0.5^a^	0.25–1.0	0	0	0.25^a^	0.25
Amp. B	*C. parapsilosis*	0,5^a^	0.0625–0.5	0.5^a^	0.0625–0.5	0.25^a^	0.25–0.5	0.5^a^	0.25–0.5
	*C. krusei*	0.5^a^	0–32	0.25^a^	0.25–32	0.5^a^	0.5	0.5^a^	0.5
	*C. tropicalis*	0.5^a^	0.25–0.5	0.5^a^	0.25–1.0	-	-	-	-
	*C. lusitaniea*	0.5^a^	0.5	0.5^a^	0.5	-	-	-	-
	*C. albicans*	32^d^	0.0625–32	32^d^	0.125–64	0.125^a^	0–0.125	64^d^	0.25–64
	*C. glabrata*	0.5^a^	0.125–1.0	2.0^a^	0.25–64	0	0	0.25^a^	0.25
KET	*C. parapsilosis*	0.5^a^	0.125–0.5	1.0^a^	0.5–32	0.125^a^	0–0.5	1.0^a^	0.25–4
	*C. krusei*	0	0–32	0	0–32	0	0	0	0
	*C. tropicalis*	4.0^d^	0–16	2.0^d^	2.0–64	-	-	-	-
	*C. lusitaniea*	0.5^a^	0–1.0	4.0^d^	0–32	-	-	-	-

^a^susceptible, ^b^susceptible dose-dependent, ^c^intermediate resistance, ^d^resistance; MIC_90_ Inhibition = 90 % of isolates

A variation between the maximum and minimum values of MIC for each species for both individuals test group as in the control group was observed. The MIC_90_ values represent the concentration of the antifungal drug that inhibited the growth of 90 % of isolates. The data were computed as the concentrations of antifungal agent necessary to inhibit 90 % of the isolates (MIC_90_) of the yeasts. *C. albicans* strains from both cancer patients and from the control group taken together gave an MIC_90_ value for amphotericin B, fluconazole and voriconazole of 64 μg/mL. For isoconazole, the MIC_90_ values for the yeast strains isolated from orogastric cancer patients and fromthe control group were 16 μg/mL and 32 μg/mL, respectively. In addition, The MIC_90_ value for *C. albicans* with ketoconazole was 1 μg/mL for the two evaluated groups. For non-*albicans* yeast isolated from patients with orogastric cancer, the MIC_90_ values for amphotericin B ranged from 1-64 μg/mL, for fluconazole it was 64 μg/mL, for isoconazole it ranged from 16-64 μg/mL, for ketoconazole it was 0.5 μg/mL and for voriconazole it ranged from 1.8-64 μg/mL. In addition, for non-*albicans* strains isolated from healthy individuals, the MIC_90_ value for amphotericin B and fluconazole was64 μg/mL. Moreover, the MIC_90_ values for voriconazole, isoconazole and ketoconazole were ≥ 0.5, ≥4 and 1 μg/mL, respectively.

### Serum cytokine detection

**Fig. 1:**
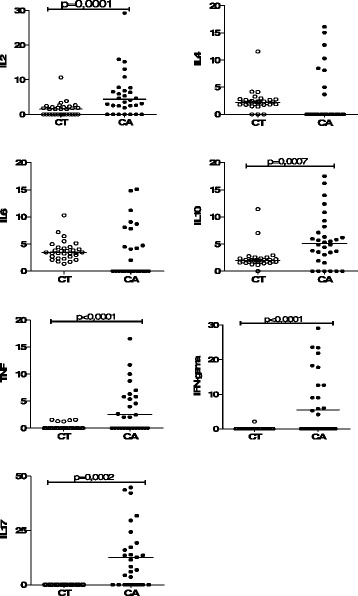
Panel measured cytokines in the serum of control subjects (CT = 31) and patients with cancer (CA = 33). Average between the existing values. IL-2 *p* = 0.0001, IL-10 *p* = 0.0007, *p* = 0.0001 TNF-α, IFN-γ *p* = 0,0001 e IL-17 *p* = 0,0002

Serum cytokine levels in patients with orogastric cancer and control group are shown in Table 3 and Fig. 1. According to the results, a statistically significant increase of IL-2, IL-10, TNF-α, IFN-γ, and IL-17 was observed in patients with cancer compared with the control group. IFN-γ was detected in 57.6 % of study cases and in 6.45 % of the control cases. The mean value of serum IFN-γ levels in patients from the study group (patients with orogastric cancer) was 18.2 ± 32.28 ρg/mL. In the control group, the mean IFN-γ value was 0.77 ± 3.92 ρg/mL. The mean value of serum IFN-γ levels in the study group was significantly different from that in the control group (*p* = 0.0001).

**Table 3:** Serum levels of cytokines (ρg/mL) in the investigated individuals

	Patients with cancer					Control group				
	N	Detectability	Min	Max	Mean ± SD	N	Detectability	Min	Max	Mean ± SD
IFN-γ	19	57.6 %	0	149.39	18.2 ± 32.28	2	6.45 %	0	21,81	0.77 ± 3.92
TNF-α	20	60.6 %	0	93.69	9.71 ± 19.06	5	15.15 %	0	1.55	0.22 ± 0.53
IL-2	26	78.8 %	0	93.03	10,71 ± 19.31	17	54.8 %	0	10.66	1.48 ± 2.08
IL-4	13	39.4 %	0	128.81	11.04 ± 26.23	28	90.3 %	0	11.57	2.42 ± 1.94
IL-6	16	48.5 %	0	219.16	16.97 ± 43.99	31	100 %	0	10,29	3.78 ± 1.79
IL-10	27	81.8 %	0	91.44	8.66 ± 15.84	30	96.8 %	0	11.43	2.36 ± 1.99
IL-17	23	69.7 %	0	356.21	33.36 ± 68.29	5	16.12 %	0	1.55	0.60 ± 3.37

*N* number of individual

TNFα was detected in 60.6 % of the patients and in 16 % of the individuals from the control group. The mean value of serum TNFα levels in patients from the study group (patients with orogastric cancer) was 9.71 ± 19.06 pg/mL. In the control group, the mean TNFα value was 0.22 ± 0.53 ρg/mL. The mean value of serum TNFα levels in the study group was significantly different from that in the control group (*p* ≤ 0.0001).

Serum levels of IL-10 in patients from the control group was 81.8 % and 96.8 % in cancer subjects. The mean value of serum IL-10 level in the study group (patients with orogastric cancer) was 8.66 ± 15.84 pg/mL. In the control group, the mean value of IL-10 levels was 2.36 ± 1.99 ρg/mL. The mean value of serum IL-10 levels in study group was significantly different from that in the control group (*p* ≤ 0.001). Serum levels of IL-17 in individuals from the study weredetected in 69.7 % of patients in the study group and in 3.22 % of individuals in the control group. The mean value of serum IL-17 levels in patients in the study group (patients with orogastric cancer) was 33.36 ± 68.29 ρg/mL. In the control group, the mean value of IL-17 level was 0.6 ± 3.37 ρg/mL. The mean value of serum IL-17 levels in the study group was significantly different from that in the control group (*p* ≤ 0.0002).

Serum levels of IL-2 in individuals from this study were detected in 78 % of patients in the study group and in 54.8 % of the individualsinthe control group. The mean value of serum IL-2 levels in patients of the study group (patients with orogastric cancer) was 10.7 ± 19.31 ρg/mL. In the control group, the mean value of serum IL-2 levels was 1.48 ± 2.08 ρg/mL. The mean value of serum IL-2 levels in the study group was significantly different from that in the control group (*p* ≤ 0.0001).

## Discussion

Infections by *Candida* species are common in cancer patients, and the incidence has increased in recent years [34, 35]. Several virulence factors contribute to *Candida* yeasts pathogenicity, being one of the main causers of fungemia in individuals with cancer and contributing to high mortality rates. In this study, it was observed that 85 % of individuals were colonized by one or more *Candida* species, with *C. albicans* being the most constant species followed by *C. glabrata.* These results are very similar to other previous reports. In a study conducted by Galle et al. [36] it was observed that among 31 % of patients with lesions of oral cancer, strains of *C. albicans* were the most prevalent species at 43.7 %, followed by *C. glabrata* and *C. tropicalis*.

A more adherent profile was detected for *Candida* species isolated from the oral cavity of individuals with cancer when compared to the strains isolated from the control group. Adhesion is considered an extremely important virulence factor in yeast given that the colonization and infection of oral tissues is directly related to adherence competence. This virulence factor may initially contribute to tissue invasion: once adhered to initiate the invasion, the yeast secretes enzymes that damage the mucosa. The higher phospholipase activity is related to stronger adherence to epithelial cells and increased pathogenicity [9]. Phospholipases and proteases activities and adhesion of microorganisms on cell surfaces are considered important virulence factors, facilitating the establishment of infection, especially in opportunistic fungi [37]. These enzymes facilitate adherence and tissue penetration and hence, host invasion. In this study, proteinases and phospholipases were detected in 6.4 % and 37.1 % of strains of *C. albicans* and 33.8 % and 14.5 %, respectively for non-*albicans* species. Phospholipases and proteases are produced at high rates by *C. albicans*, while species of *Candida* non-*albicans* generally have low levels of these enzymes [38]. Koga- Ito et al. [39] showed an increased production of proteinases and phospholipases between strains of *C. albicans* isolated from patients with oral candidiasis when compared with those isolated from control subjects.

Numerous studies regarding the MIC of *Candida* spp. isolates are found in the literature in recent years because of the increased incidence of invasive candidiasis, along with an increase in the isolation of species resistant to antifungal drugs [40]. Patients undergoing radiotherapy for head and neck cancer represent a serious challenge in relation to oral aftercare due to radiotherapy complications [41]. However, it is important to know the susceptibility of antifungal drugs before deciding on a specific treatment because some species are intrinsically resistant to certain antifungal drugs.

In this study, amphotericin B showed good activity against yeast strains from both cancer patients and from the control group, with MIC_90_ values of ≤1 μg ml/mL for all major isolates. In fact, all the isolates were susceptible, except for *C. krusei.* Only a few of *Candida* isolates (6.5 %) were resistant to isoconazole, and 8 % of *Candida* isolates showed resistance to voriconazole. However, 35 % of the yeasts from patients with cancer were resistant to fluconazole. This is of concern given that fluconazole has been used as a first line therapy for infections caused by *Candida* species other than *C. glabrata* and *C. krusei* [42]*.*

In the present study, we observed that in patients who had orogastric cancer, inflammatory cytokines showed statistically significant increases compared to the control group (individuals considered healthy), showing agreement with the literature. Cancer development from the inflammatory process can be conducted by tumor-associated macrophages*,* as well as a variety of chemical mediators [43, 44]. More recently, inflammation has been shown to be a critical component in tumor progression [45]. Moreover, the emergence of several types of cancer can be observed at sites of infection and inflammation [45]. Immune cells and regulatory molecules secreted by these cells within the tumor microenvironment have key roles in antitumor immunity and evasion of the immune system [44, 46].

Previous studies in vitro with human cells and squamous cell carcinoma showed that the concentration of certain pro-inflammatory cytokines and pro-angiogenic cytokines, such as TNF-α*,* IL-1, IL-6 and IL-8, are increased. There is evidence that cytokines are produced in an unregulated way in oropharyngeal cancer and that they have roles in cellular invasion, interruption in suppressing tumor growth and immunological status [47, 48]. In this study, individuals with orogastric cancer also showed elevated levels of TNF-α, showing a close relationship with this type of tumor.

We observed that individuals with orogastric cancer also showed a high levels of IL-10 in their serum relative to the control group, showing agreement with other studies. It has been reported that IL-10 plays a role in carcinogenesis, which is considered controversial, in both promotion and inhibition of tumors [49]. In colorectal carcinogenesis, IL-10 promotes cancer growth, rather than inhibition, through its immunosuppressive activity [50, 51]. Previous studies have suggested that the increase in IL-10 can track inflammatory responses and cancer development and constitute a risk factor for carcinogenesis [51].

We also quantified Interleukin 17 (IL-17) in the serum of individuals with and without cancer and statistically significant differences were detected. Our data suggest that the immune status of these individuals was changed. Interleukin 17 (IL-17)-mediated immunity plays a key role in protection from fungal infections in mice and man [52, 53]. Here, we confirmed that mice deficient in IL-17 receptor or lacking the ability to secrete IL-17 are highly susceptible to systemic candidiasis [52]. Individuals with cancer investigated in the present study had no apparent candidiasis injury because collection was performed before the initiation of treatment, after confirmation of the diagnosis. However, given the existing changes in the immune system of these individuals, they were susceptible to opportunistic infections, such as candidiasis.

Gasparoto et al. [54] showed, in elder individuals, showed that the immunoregulatory cytokine IL-10 has the ability to inhibit IL-12 and IFN-γ, promoting an immune response type II and show that this type of response (Th2) is associated with the susceptibility of the host to infection by *C. albicans* [43]. According XIONG et al [55], the virulence of *C. albicans* may be related to its ability to selectively induce IL-10, with simultaneous inhibition of IL-12 and T cells IFN-γ allowing a greater susceptibility of the individual candidiasis [44]. Then, one can also conclude that early monitoring of levels of interleukins, in particular IL-10, may contribute tomaking clinical decisions regarding the prophylaxis forfungal infections subsequent or concomitant tothe treatment of malignant tumors.

## Conclusion

It was possible to isolate *Candida* species in 85 % and 59 % of individuals with COG-C and in the control group, respectively. The species *C. albicans* was the most prevalent amidst the isolates. As for virulence factors, the isolates obtained from patients with cancer were more virulent than isolates from the control group. The results generated by MIC assessment showed that the isolates were susceptible to all antifungals compounds. The 20 drugs to which the yeast strains presented higher susceptibility were amphotericin B, ketoconazole and isoconazole. Regarding the cytokines evaluated in the serum of individuals with and without cancer, significant differences were observed among individuals with cancer for IL2, IL10, TNF-α, IFN-g and IL17.

## Abbreviations

AmpB: amphotericin B
BEC: buccal epithelial cells
CBA: cytometric bead array
FLU: fluconazole
HBECs: human buccal cells
IFN-γ: interferon
IL-10: interleukin 10
IL-17: interleukin 17
IL-2: interleukin 2
ISO: isoconazole
KET: ketoconazole
MALDI-TOF: matrix assisted laser desorption ionization – time of flight
MG: Minas Gerais
MIC: minimum inhibitory concentration
MS: mass spectrometry
NaCl: sodium chloride
OGC: orogastric cancer
PZ: phospholipases
Sap: aspartyl proteinases
SDA: sabouraud dextrose agar
TNF-α: tumor necrosis factor
VOR: voriconazole.
YCa: yeasts cancer
YCo: yeasts control
